# Uncommon pathogen, common activity: *Streptococcus pneumoniae* conjunctivitis outbreak traced to a primary school field trip

**DOI:** 10.3389/fpubh.2026.1893282

**Published:** 2026-09-01

**Authors:** Xiaojian Duan, Xiaobin Ren, Kai Song, Jiayun Wang, Yi Wang, Yuanming Zhang, Li Ren, Liangliang Huo, Jing Wang

**Affiliations:** 1 Hangzhou Center for Disease Control and Prevention (Hangzhou Health Supervision Institution), Institute of Infectious Disease Control and Prevention, Hangzhou, China; 2 Qiantang District Center for Disease Control and Prevention, Institute of Infectious Disease Control and Prevention, Hangzhou, China

**Keywords:** bacterial conjunctivitis, conjunctivitis, educational field trip, epidemiological traceability investigation, outbreak, primary school, *streptococcus pneumoniae*

## Abstract

**Background:**

In December 2025, an outbreak of acute conjunctivitis emerged at a primary school in Hangzhou, China, affecting 48 students and yielding an attack rate of 2. 16%. The outbreak is of particular significance as it was attributed to *Streptococcus pneumoniae*, a pathogen seldom implicated in large-scale conjunctivitis outbreaks among school-aged children.

**Methods:**

An epidemiological investigation, a case-control study, and laboratory analyses—comprising multiplex real-time PCR and bacterial culture—were conducted to determine the etiological agent and the source of transmission.

**Results:**

A significant association was established between the outbreak and a recent educational field trip (OR = 6.42, 95% CI: 3.53–11.68), suggesting that such organized group activities may represent an important transmission setting for bacterial conjunctivitis. Laboratory analyses confirmed the presence of *S. pneumoniae* in conjunctival swabs and environmental samples collected from the field trip venue. Moreover, students treated with combination antibiotic therapy had a significantly shorter mean return-to-school time compared with those receiving monotherapy or no treatment (*p* < 0.01). However, as treatment allocation was not randomized, this association should be interpreted as an observational finding rather than evidence of causal effectiveness.

**Conclusion:**

This investigation underscores the distinct transmission risks inherent to field trips and emphasizes the imperative of embedding comprehensive infection prevention and control measures into the foundational planning of such activities.

## 1 Introduction

Acute conjunctivitis is a prevalent ocular infection globally, presenting with conjunctival injection, edema, increased exudate, and ocular discomfort (1, 2). Highly transmissible, it readily triggers outbreaks in crowded environments, imposing a significant public health and socioeconomic burden. Worldwide, its incidence fluctuates according to geographic region, seasonality, and population demographics. In temperate regions, viral conjunctivitis typically peaks during summer and autumn, whereas in tropical regions, it may persist endemically throughout the year (3–5). Incidence is markedly elevated among children, adolescents, and populations in congregate settings—such as students, military personnel, and factory workers—largely owing to close interpersonal contact and frequent sharing of personal items (6, 7).

Etiologically, acute conjunctivitis arises from viral, bacterial, or allergic causes. Viral etiology underlies over 80% of large-scale outbreaks (8, 9). Adenoviruses, particularly serotypes 8, 19, and 37, are the principal agents responsible for viral conjunctivitis outbreaks, manifesting as pharyngoconjunctival fever or epidemic keratoconjunctivitis. These viruses exhibit high transmissibility and can precipitate outbreaks in schools, swimming pools, and healthcare settings (10, 11). Furthermore, enterovirus 70 and the coxsackievirus A24 variant are the principal causative agents of global acute hemorrhagic conjunctivitis pandemics (12, 13). By contrast, bacterial conjunctivitis typically presents as sporadic cases, with common pathogens including Haemophilus influenzae, Moraxella catarrhalis, and Staphylococcus aureus (14–16). *Streptococcus pneumoniae* contributes to 2.7%−41.2% of bacterial conjunctivitis cases, with a predominance in infants and young children; it is less prevalent among school-aged children and seldom implicated in large outbreaks.

Current evidence indicates that outbreaks of *S. pneumoniae* conjunctivitis predominantly arise within semi-closed environments, including community settings, university dormitories, and military barracks (17, 18). Although several *S. pneumoniae* conjunctivitis outbreaks have been reported in university campuses, military barracks, communities, and prisons, none of these studies identified an off-campus educational field trip as the common exposure. A targeted literature search yielded no prior reports linking such organized school excursions to pneumococcal conjunctivitis clusters. In contrast to the swift dissemination characteristic of viral conjunctivitis, bacterial conjunctivitis typically exhibits slower transmission, accompanied by distinct incubation periods and clinical manifestations. These features may result in delayed detection of outbreaks and postponed implementation of control measures (19). The incubation period is a critical epidemiological parameter for understanding outbreak dynamics. For bacterial conjunctivitis caused by *S. pneumoniae*, the incubation period is typically 1–3 days after exposure (19). This relatively short and narrow window means that cases arising from a common exposure tend to cluster within a few days, facilitating the identification of point-source outbreaks. By contrast, the longer and more variable incubation period of viral conjunctivitis—ranging from 1 to 12 days for adenoviruses—can obscure the timing of exposure and make source identification more challenging (9). The distinct incubation profile of *S. pneumoniae* thus has important implications for outbreak detection and source tracing: when cases cluster tightly in time following a shared exposure, a bacterial point-source should be suspected, prompting rapid environmental and microbiological investigation. Nonetheless, the consequences of such outbreaks warrant serious consideration when transmission occurs among densely concentrated susceptible populations, such as those in primary schools. To date, comprehensive and systematic analyses detailing the epidemiological features of *S. pneumoniae* conjunctivitis outbreaks remain scarce, and no study has specifically investigated their association with short-term, organized off-campus group activities such as educational field trips, which represent a distinct transmission setting.

School environments represent established venues for outbreaks of respiratory and contact-transmitted diseases (20, 21). Recent studies have provided quantitative insights into the factors that drive infectious disease transmission within schools. Prolonged exposure in shared, poorly ventilated classrooms has been identified as a stronger driver of respiratory virus transmission than close-proximity interactions alone, with time in shared classrooms associated with an approximately nine-fold higher transmission risk (22). Indoor environmental quality plays a critical role: more than one-third of illness-related absences could potentially be reduced by upgrading school ventilation and maintaining recommended standards for surface cleanliness (23). High-touch surfaces such as desks, door handles, and faucets serve as environmental sources of pathogens, and transmission can occur when contaminated hands contact mucous membranes. These findings underscore the importance of both environmental hygiene and building infrastructure in school-based outbreak prevention.

Beyond the school premises, organized off-campus group activities introduce additional transmission risks that differ from routine school exposure. Field trips and study tours involve prolonged time in enclosed spaces (e.g., buses, dormitories), shared lodging and dining facilities, close physical collaboration during team activities, and a potential relaxation of personal hygiene practices in unfamiliar settings. These conditions can facilitate both direct person-to-person and fomite-mediated transmission (21). Evidence from a COVID-19 outbreak following a high school field trip in the Republic of Korea reported an attack rate of 25.8% among participants, with field trip attendance associated with a more than two-fold increased odds of infection (OR = 2.39, 95% CI: 1.66–3.43) (24). Similarly, a Shiga toxin-producing E. coli O157:H7 outbreak among elementary school students was traced to a farm animal exhibit field trip, affecting approximately 2300 attendees and resulting in hospitalisations (25). These examples illustrate that field trips can serve as effective transmission “triggers” for diverse pathogens, yet systematic evidence linking such activities to bacterial conjunctivitis outbreaks—particularly those caused by *S. pneumoniae*—remains sparse.

In December 2025, an anomalous cluster of acute infectious conjunctivitis cases emerged within a primary school in Hangzhou, China. The outbreak progressed swiftly, impacting multiple classes over a brief interval. Initial inquiries excluded adenovirus as the causative agent, thereby redirecting investigative attention toward bacterial etiologies. To guide this investigation, we formulated the following research questions and hypotheses: Research Question 1: What was the etiological agent responsible for this acute conjunctivitis outbreak? Research Question 2: What was the source of transmission, and could the outbreak be linked to a specific exposure event?

Research Question 3: What were the epidemiological and environmental factors that facilitated transmission? Hypothesis: We hypothesized that the outbreak was caused by a bacterial pathogen, likely introduced during a recent off-campus educational field trip, with subsequent person-to-person transmission within the school. To identify the aetiological agent, determine the source of transmission, and characterize the epidemiological features of the outbreak to inform targeted infection prevention and control measures.

## 2 Materials and methods

### 2.1 Study subjects

Suspected Case: Any school staff member or student, since November 20, 2025, exhibiting one or more of the following symptoms—conjunctival injection, ocular pain, foreign body sensation, photophobia, epiphora, ocular burning, blurred vision, eyelid edema, or ocular discharge—with alternative definitive diagnoses having been ruled out.

Clinically Diagnosed Case: A suspected case manifesting two or more of the aforementioned symptoms.

Confirmed Case: A suspected or clinically diagnosed case in which a conjunctival swab yields a positive result for *Streptococcus pneumoniae* via real-time fluorescent quantitative polymerase chain reaction (real-time PCR).

### 2.2 Case ascertainment

Case identification was conducted in strict adherence to the established case definition, drawing upon a multi-faceted approach that included the review of school clinic records from morning and noon inspections, an analysis of student absenteeism logs, and targeted inquiries into the occurrence of similar symptoms among close contacts. Additionally, homeroom teachers were tasked with conducting comprehensive, class-wide assessments of student health status.

### 2.3 On-site hygienic survey

An on-site inspection was carried out to evaluate the school's structural design—encompassing classrooms and the cafeteria—as well as environmental cleanliness, alongside the safety and sanitation standards of food and water supplies.

### 2.4 Case-control study

Guided by risk factor hypotheses derived from descriptive epidemiology and field surveys, an exposure assessment was conducted among cases and controls. Odds ratios were subsequently calculated to identify potential drivers of the outbreak. Controls were selected from the same school population as the cases, comprising all students who did not meet the case definition during the outbreak period. Individual matching by age, class, or sex was not performed to avoid overmatching, as the primary exposure (field trip participation) was a school-wide activity with uneven distribution across grades.

### 2.5 Laboratory testing

All collected conjunctival swabs and environmental surface samples were transported on ice and processed immediately upon arrival at the laboratory, within 2 h of collection. Environmental surface sampling was conducted by trained CDC investigators wearing sterile gloves. Sterile bacterial transport swabs with flocked tips and plastic shafts, pre-filled with transport medium (buffered peptone water or sterile saline), were used for all environmental collections. For each sampling site, the swab tip was moistened with the transport medium and used to sample a standardized area of approximately 100 cm^2^ (10 cm × 10 cm) using a systematic back-and-forth motion (10 vertical, 10 horizontal, and 10 diagonal strokes) while rotating the swab. The following surfaces were sampled: classroom desks, door handles, faucet handles, shared educational equipment (at the school); and bed rails, sink handles, door handles, and dining table surfaces (at the field trip base). After collection, each swab was immediately inserted back into its sterile transport tube; the cap was securely tightened, and the tube was clearly labeled with the sample ID, collection date, and collector's name. Samples were transported on ice packs (2–8°C) to the laboratory within 2 h of collection. To prevent cross-contamination, personnel washed and sanitized hands before sampling, sterile gloves were changed between each sample, a new sterile swab was used for each surface, swab tips were not touched during handling, and negative field controls were included. Environmental and clinical specimens were transported separately.

#### 2.5.1 Nucleic acid extraction and preliminary pathogen screening

Total nucleic acid was extracted from each sample using a commercial extraction kit. All samples were initially screened by multiplex real-time PCR for common viral pathogens associated with acute conjunctivitis, including enteroviruses and enteric adenoviruses.

Samples that tested negative on the viral panel underwent further analysis using a commercial 22-pathogen multiplex real-time PCR kit (Meningitis/Encephalitis Syndrome 22-Pathogen Nucleic Acid Detection Kit, PCR-Fluorescence Probing; Beijing Applied Biological Technologies Co., Ltd., China), which simultaneously detects 22 bacterial, viral, and fungal pathogens associated with central nervous system infections. The kit has a limit of detection of 500 copies/mL, demonstrates no cross-reaction with non-target pathogens, and includes an internal control (RNP gene), positive control, and negative control to monitor specimen quality and reaction validity.

For conjunctival swab samples positive for bacterial nucleic acid—particularly those indicative of *S. pneumoniae*—conventional bacterial culture was performed. Samples were inoculated onto appropriate agar media, and suspected colonies were isolated and purified. Species-level identification was confirmed using matrix-assisted laser desorption/ionization time-of-flight mass spectrometry (MALDI-TOF MS) and an automated microbiology identification system.

### 2.6 Statistical analysis

Epidemiological and laboratory data were recorded and managed using Microsoft Excel 2019. All statistical analyses were conducted with SPSS version 27.0 (IBM Corp.) and GraphPad Prism version 8.0.2 (GraphPad Software), with a significance level (α) of 0.05 applied to all two-tailed tests.

Descriptive statistics were employed to characterize the outbreak. Attack rates were computed for the overall population and stratified by key variables, including class, sex, and field trip participation. The temporal pattern of the outbreak was visualized using an epidemic curve, while clinical symptoms were reported as frequencies and percentages.

In the case-control study, field trip participation served as the primary exposure of interest. Odds ratios (ORs) and 95% confidence intervals (CIs) were calculated. For continuous variables, one-way ANOVA was performed, followed by Tukey's post-hoc test. For categorical variables, the Pearson χ^2^ test was utilized; Fisher's exact test was applied where expected cell frequencies were below 5.

## 3 Results

### 3.1 General information

The subject school in Hangzhou is a non-boarding institution comprising 48 classes, with a total enrollment of 2,071 students (1,075 male and 996 female, aged 7–12 years) and a staff of 151, which included one school doctor.

### 3.2 Case findings

A total of 48 cases were identified, all among the student body, resulting in an attack rate of 2.16% (48/2222). The cases comprised 10 laboratory-confirmed cases and 38 clinically diagnosed cases.

### 3.3 Clinical manifestations

The predominant clinical symptoms among the 48 cases included conjunctival injection, ocular discharge, and a foreign body sensation, accompanied by photophobia and excessive tearing. All cases were mild in nature, with no instances of hospitalization, severe illness, or mortality (Table 1).

**Table 1:** Clinical manifestations of acute conjunctivitis cases in a school in Hangzhou, China, 2025.

Clinical symptom	Number of cases	Proportion (%)
Conjunctival injection	40	83.33
Eye discharge	33	68.75
Foreign body sensation	22	45.83
Photophobia	6	12.50
Tearing	7	14.58

### 3.4 Epidemiological characteristics

#### 3.4.1 Temporal distribution

**Figure 1:**
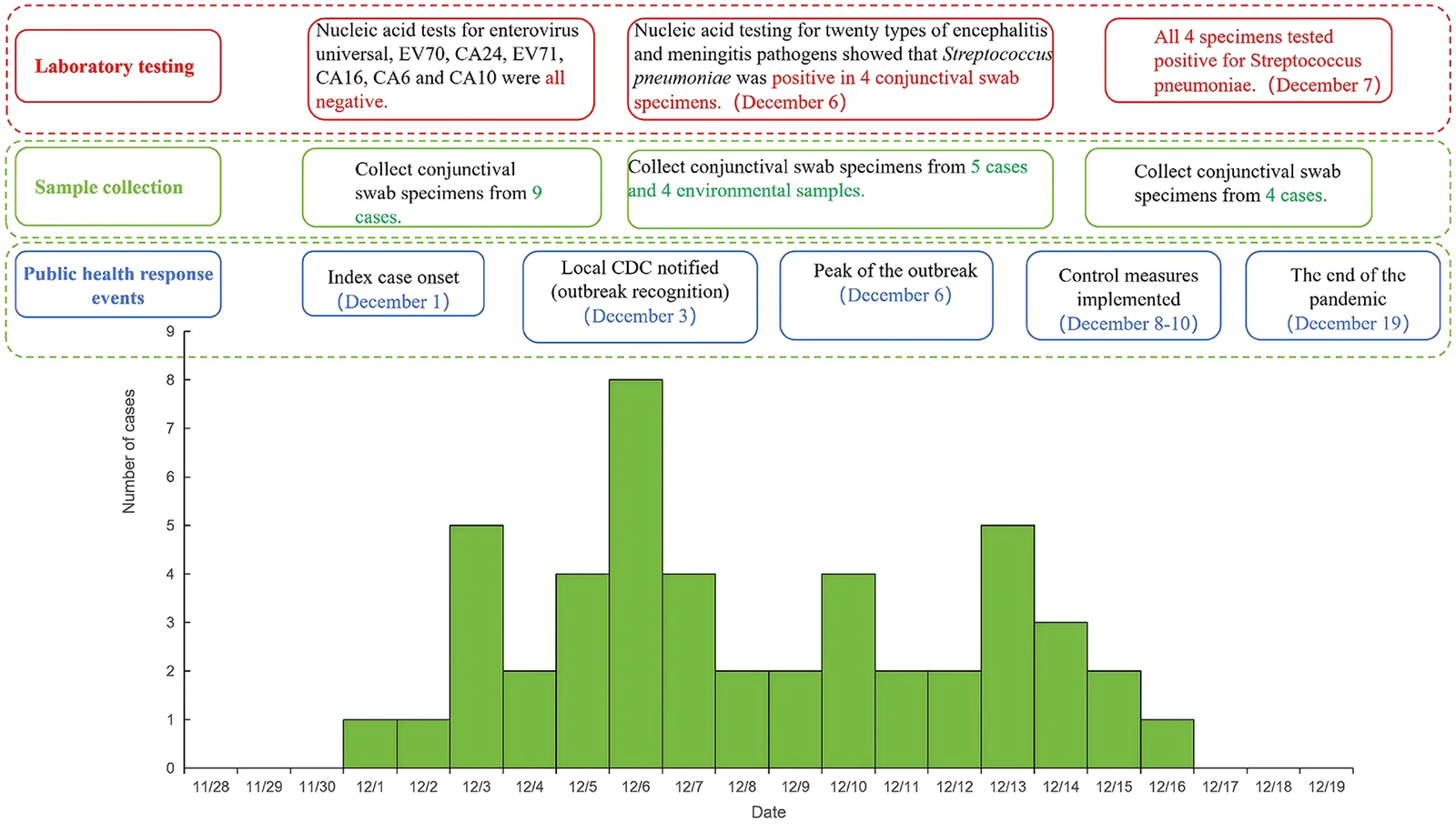
Epidemic curve of acute conjunctivitis cases (*n* = 48) by date of symptom onset, Hangzhou, China, December 2025.

The index case presented on December 1, having participated in an off-campus group field trip from November 28–30, which included overnight accommodation at the activity site. The patient sought medical care on December 2 after parents observed increased periorbital discharge and conjunctival injection. A diagnosis of acute hemorrhagic conjunctivitis was established, and treatment with ofloxacin eye drops was initiated. The patient's symptoms subsequently improved, and the individual returned to school on December 5. The final case onset occurred on December 16. The outbreak peaked on December 6 with 8 cases, and the overall duration was 16 days. The epidemic curve suggested an initial point-source exposure, followed by subsequent person-to-person transmission (Figure 1).

#### 3.4.2 Spatial distribution

Cases were reported across 33.33% (16/48) of the school's classes. The fourth grade accounted for the majority of cases, at 60.42% (29/48), with class 405 reporting the highest incidence (15 cases). Nine cases occurred in the fifth grade, while the remaining cases were distributed among the first and second grades. The attack rate varied significantly among classes (χ^2^ = 42.24, *p* < 0.01).

Cases were predominantly located on the second and third floors, which together represented 79.17% (38/48) of the total. The attack rate was 3.31% on the second floor (11 cases) and 6.80% on the third floor (27 cases). This distribution of attack rates across floors also differed significantly (χ^2^ = 24.81, *p* < 0.01) (Table 2).

**Table 2:** Stratified analysis of attack rates for acute conjunctivitis cases in a school in Hangzhou, China, 2025.

Category		Cases	Attack rate (%)	χ^2^	*p*
Sex	Male	32	2.98	4.29	0.04
	Female	16	1.61		
Age	6–8 years	8	1.05	8.67	< 0.001
		9–11 years	40	3.06	
Grade	1	4	0.81	42.24	< 0.001
		2	4	0.80	
		3	29	6.67	
		4	10	2.37	
Floor	1st	6	1.97	24.81	< 0.001
		2nd	11	3.31	
		3rd	27	6.80	
		4th	4	0.92	

#### 3.4.3 Demographic distribution

There were 32 cases among males, with an attack rate of 2.98% (32/1075), compared to 16 cases among females (attack rate 1.61%, 16/996). This difference in attack rate by sex was statistically significant (χ^2^ = 4.29, *p* = 0.04). The majority of cases, 80.33% (40/48), were concentrated in the 9–11-year age group. The age of cases ranged from 6 to 11 years, with a median age of 10. Attack rates also differed significantly by age group (χ^2^ = 8.67, *p* < 0.001) (Table 2).

#### 3.4.4 Laboratory findings

A total of 18 conjunctival swabs and 8 environmental surface samples were collected and tested. PCR analysis identified *Streptococcus pneumoniae* in 9 of the conjunctival swabs, yielding a positivity rate of 50.0% (9/18). Similarly, 4 environmental samples tested positive, with a positivity rate of 50.0% (4/8). Bacterial culture was subsequently performed on four of the PCR-positive conjunctival swabs, and identification of the purified colonies by MALDI-TOF MS confirmed the presence of *S. pneumoniae* with 95% probability. The 4 selected specimens were chosen based on the following criteria: they were collected from patients with active symptomatic disease who had not received prior antibiotic treatment. The remaining 5 PCR-positive specimens were not cultured because the patients had already received topical antibiotics or were in the convalescent phase with improving symptoms, which significantly reduces culture yield. This selective approach was consistent with the primary objective of confirming the etiological agent identified by PCR; once *S. pneumoniae* was successfully cultured from four independent specimens, the etiological confirmation was considered definitive. Culturing additional specimens would have provided diminishing returns while consuming limited laboratory resources during the emergency response. All 8 environmental samples (four from the school and four from the field trip base) were also subjected to bacterial culture on Columbia blood agar under 5%−10% CO_2_ at 37 °C for 24–48 h; however, no *S. pneumoniae* was isolated from any environmental sample. This outcome is consistent with the known environmental fragility of *S. pneumoniae*, which rapidly loses viability on desiccated surfaces, and with the higher sensitivity of PCR compared with culture.

### 3.5 Medication pattern analysis

**Figure 2:**
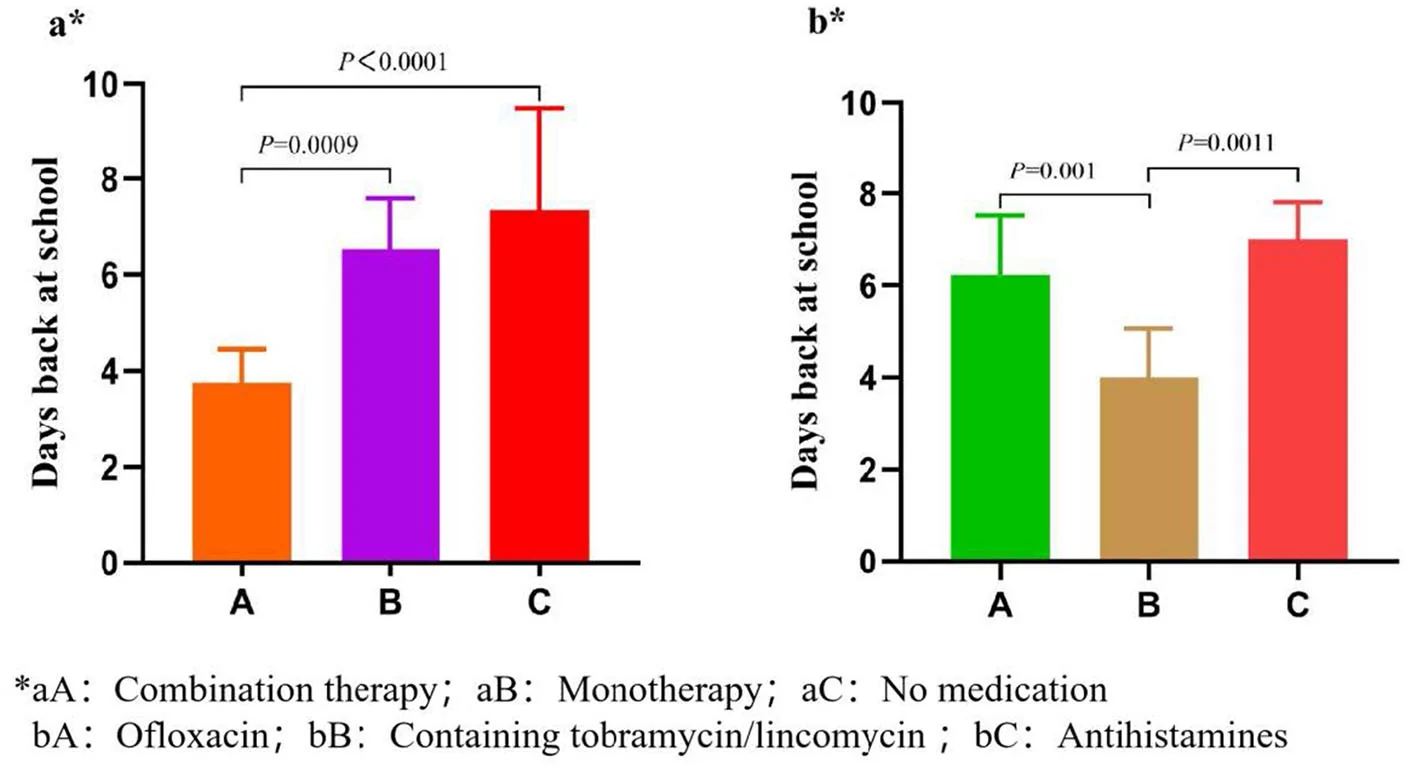
Comparison of return-to-school time among students, stratified by medication type.

Of the 48 cases, 25 had a documented medical visit history. To aid in pathogen determination, we analyzed the association between medication type and regimen and the time required for students to return to school. As treatment allocation was not randomized, this analysis is observational and does not establish causality. A significant difference in return time was observed among students receiving combination therapy, single-drug therapy, and no medication (F = 14.08, *p* < 0.001). *Post-hoc* tests revealed significant differences between the combination therapy group and both the single-drug and no-medication groups (*p* < 0.01), whereas no significant difference was found between the single-drug and no-medication groups (*p* = 0.28) (Table 3, Figure 2).

**Table 3:** Analysis of the number of days until return to school, stratified by medication type, for cases of acute conjunctivitis in Hangzhou, China, 2025

Medication category	Mean return days (±SD)	F-statistic	*p*-value
Combination therapy	3.75 (±0.75)	14.08	< 0.001
Single-drug therapy	6.53 (±1.07)		
No medication	7.35 (±2.15)		
Ofloxacin	6.23 (±1.30)	12.14	< 0.001
Tobramycin/lincomycin	4.00 (±1.70)		
Antihistamine	7.00 (±0.82)		

Similarly, significant differences in return time were observed among users of ofloxacin, tobramycin/lincomycin-containing drops, and antihistamines (F = 12.14, *p* < 0.001). *Post-hoc* analyses indicated significant differences between the tobramycin/lincomycin group and both the ofloxacin and antihistamine groups (*p* < 0.001), but not between the ofloxacin and antihistamine groups (*p* = 0.50) (Table 3, Figure 2).

### 3.6 Investigation of related factors

#### 3.6.1 Outbreak source investigation

The local CDC was notified on December 3. Conjunctival swabs from nine cases with an onset on that day were tested for EV70, CA24v, EV71, CA16, CA6, CA10, and enteric adenoviruses; all results were negative.

The epidemiological investigation revealed a concentration of cases within the fourth grade, located on the third floor. School officials reported that the fourth-grade students had attended a field trip to a rural base the week prior to the index case's onset, during which they shared meals and accommodation. Based on the initial laboratory findings and epidemiological evidence, a bacterial etiology was suspected. On December 6, five conjunctival swabs and four classroom surface samples were collected. Testing using a 22-pathogen PCR kit identified *S. pneumoniae* nucleic acid in all five conjunctival swabs, while all environmental samples tested negative.

On December 7, environmental samples were collected from four student dormitories at the field trip base (including bed rails, sinks, and door handles). All four samples tested positive for *S. pneumoniae* nucleic acid, suggesting that the field trip base was the likely source of the outbreak, followed by subsequent person-to-person transmission within the school.

#### 3.6.2 Case-control study

Using participation in the field trip as the exposure, the case-control study identified 29 cases among 435 participants (attack rate 6.67%) and 18 cases among 1,636 non-participants (attack rate 1.10%). This association was statistically significant (OR = 6.42, 95% CI: 3.53–11.68), supporting the field trip as the source of the outbreak (Note: One of the 48 cases could not be definitively classified regarding field trip participation and was excluded from this analysis). Multivariable adjustment was precluded by the unavailability of individual-level control data; however, the magnitude and precision of the effect estimate, together with the supportive epidemiological evidence, strongly indicate that confounding is unlikely to explain the finding.

#### 3.6.3 School hygiene, food, and water supply investigation

The school utilizes a centralized municipal tap water supply, and no consumption of untreated water was reported. Water quality is assessed each semester, with no significant abnormalities ever recorded. The facility has 38 flush toilets, which are cleaned twice daily. Waste is managed through septic tanks connected to the municipal sewer system, and garbage is collected daily by municipal services.

On weekdays, the school provides lunch exclusively through a designated cafeteria. Students are assigned fixed seats by class, and food is served by certified staff who undergo daily health monitoring; no abnormalities were detected.

However, key hygiene weaknesses were identified: (1) Disinfection of high-touch surfaces (e.g., desks, door handles, and faucets) in affected classrooms was substandard, as staff were unaware of the proper disinfectant concentration and required contact time. (2) Younger students had not yet established proper handwashing habits or the practice of avoiding eye rubbing, and staff supervision of personal hygiene was inadequate.

#### 3.6.4 Student contact patterns

The after-school care program was held daily from 4:00 PM to 6:00 PM. This program mixed students from various grades and classes, thereby creating a substantial risk for cross-transmission.

## 4 Discussion

This study documents an acute conjunctivitis outbreak caused by *Streptococcus pneumoniae*, directly linked to a primary school field trip. The outbreak's epidemiological profile, causative agent, and transmission context differ markedly from most documented acute conjunctivitis outbreaks, underscoring its unique nature and significant public health implications.

Compared with previously reported *S. pneumoniae* conjunctivitis outbreaks, the present event exhibits distinctive features in attack rates, transmission patterns, and environmental evidence. A systematic review reported a median outbreak attack rate of 7.0% (26), our overall attack rate of 2.16% falls at the lower end of this range. Regarding transmission, most previous outbreaks were associated with sustained crowding in universities, military barracks, or prisons (18). By contrast, our outbreak is distinguished by an ‘activity-triggered' model, where transmission originated from a short-term, structured off-campus field trip followed by secondary school-based spread. In contrast to a 2003 US community outbreak involving multiple families caused by non-typeable *S. pneumoniae* and a 2006 Minnesota community outbreak (18), this outbreak is distinguished by its population (concentrated among a single primary school's children), triggering scenario (a specific short-term off-campus activity), and focalized transmission (rapid spread within the school). While previous outbreaks highlighted widespread community contact, this study identified a highly structured, time-limited collective activity as the transmission “trigger.”Finally, while environmental sampling has rarely been reported in prior outbreaks, our PCR-positive results from the field trip dormitories provide direct evidence of environmental contamination at the exposure site, underscoring the role of fomites in this transmission setting.

The discrepancy between environmental sample results from the school (negative) and the field trip dormitories (positive) can be explained by several factors. First, sampling timing differed: the field trip base samples were collected from the exact exposure site where students had stayed, and these surfaces had not been subjected to the intensive cleaning that occurred at the school after the outbreak was identified. Second, environmental conditions at the dormitories (higher occupancy density, limited ventilation, and presence of organic material) may have favored bacterial persistence. This is consistent with recent evidence demonstrating that *S. pneumoniae* is desiccation-tolerant and can survive on dry surfaces for extended periods (27) and that PspA-mediated aggregation in mucosal secretions protects the organism against desiccation on fomites (28). The presence of organic material, such as respiratory droplets and skin cells, may further enhance survival by providing a protective matrix. Third, the specific surfaces sampled at the base (bed rails, door handles, and sink handles) represent high-touch fomites that are recognized as important vehicles for contact transmission. Collectively, these factors explain why environmental samples from the exposure site remained PCR positive even after one week, while school samples collected later in the outbreak investigation were negative.

The relative contribution of different transmission routes cannot be precisely quantified, but the evidence suggests a multifactorial pathway. Direct person-to-person contact was likely the primary driver of secondary transmission, given close student interactions and the epidemic curve pattern. Contaminated fomites (e.g., desks, door handles, faucets) probably played an important role, supported by PCR-positive environmental samples at the field trip dormitories and the observed eye-rubbing behavior. Shared facilities—including dormitories, dining areas, and after-school care programs—may have amplified transmission by mixing students from different classes. Environmental surfaces in the school, although PCR-negative at sampling, cannot be ruled out, as sampling occurred after intensified cleaning and disinfection. We hypothesize that the field trip introduced the pathogen into a susceptible population, followed by sustained person-to-person and fomite-mediated spread within the school.

Globally, over 80% of acute conjunctivitis outbreaks are viral, primarily driven by adenoviruses and enteroviruses such as coxsackievirus A24 variant and enterovirus 70, which are typical agents of large clusters in schools and swimming pools (29). Bacterial conjunctivitis, in contrast, is typically sporadic, with common pathogens including Haemophilus influenzae, Moraxella catarrhalis, and Staphylococcus aureus (30, 31). While *S. pneumoniae* is a recognized cause of conjunctivitis, it is infrequently identified in school-aged children, and large-scale outbreaks are rare (32, 33). Previous reports of *S. pneumoniae* conjunctivitis outbreaks have been confined to semi-closed settings with adult or mixed-age populations, such as communities, university dormitories, or military barracks (19, 34). This outbreak, characterized by the rapid transmission of *S. pneumoniae* within a dense population of primary school children, highlights the high transmissibility of bacterial pathogens in this age group. This finding stands in stark contrast to the literature's emphasis on viral dominance and the sporadic nature of bacterial infections, particularly *S. pneumoniae*. Unlike all previously reported *S. pneumoniae* conjunctivitis outbreaks, which were associated with sustained crowding in residential or communal environments, the present outbreak is distinct in both population (exclusively primary school-aged children) and triggering scenario (a time-limited field trip with shared transport and lodging). Consequently, this event strongly suggests that under specific transmission conditions, pathogens traditionally considered “minor” or “sporadic” can emerge as primary drivers of cluster infections, thereby broadening the scope of etiological considerations in school-based public health emergencies.

Our results indicated that students receiving combination therapy had a significantly shorter mean return-to-school time (3.75 ± 0.75 days) compared to those in the single-drug therapy group (6.53 ± 1.07 days) and the no-medication group (7.35 ± 2.15 days) (*p* < 0.01). This finding suggests that combination therapy—typically the concurrent use of multiple antibiotic eye drops—exerts a more rapid and comprehensive effect in controlling bacterial infection and mitigating the inflammatory response (35). During the early phase of the outbreak, before the pathogen was identified, clinicians faced considerable pressure and likely opted for more aggressive combination therapy to rapidly control symptoms and interrupt transmission. Although this study did not directly assess the pathogen clearance rate, the reduction in return-to-school time, as a key functional outcome, likely reflects faster alleviation of clinical symptoms, thereby facilitating earlier compliance with return-to-school health criteria. However, this practice raises important considerations regarding rational antimicrobial use. While the widespread adoption of combination therapy in the absence of a confirmed pathogen may shorten the disease course for individuals, this must be weighed against the risks of selective pressure, potential adverse reactions, and increased medical costs (36). Our observational data also suggest that combination antibiotic therapy was associated with a shorter return-to-school time; however, this finding requires confirmation in randomized controlled studies, as treatment allocation was not controlled and confounding by indication cannot be excluded (37).

We acknowledge that the observed association between combination antibiotic therapy and shorter return-to-school time should be interpreted with caution. Because treatment decisions were made by clinicians based on clinical presentation and evolving outbreak information, treatment allocation was not randomized. This introduces the potential for confounding by indication—for example, patients with more severe symptoms may have been more likely to receive combination therapy, or conversely, those who received combination therapy may have differed from others in ways that also influenced recovery (e.g., earlier presentation, better adherence). We did not systematically collect data on disease severity, time from onset to treatment, or treatment adherence, precluding adjustment for these potential confounders. Therefore, the observed association should be considered exploratory and hypothesis-generating rather than definitive evidence of causal effectiveness. Randomized controlled trials are needed to establish the comparative efficacy of different antibiotic regimens for *S. pneumoniae* conjunctivitis in this population.

Analysis of specific antibiotics revealed that students treated with tobramycin or lincomycin had the shortest return-to-school time (4.00 ± 1.70 days). No significant difference was observed between users of ofloxacin (6.23 ± 1.30 days) and antihistamines (7.00 ± 0.82 days). Tobramycin (an aminoglycoside) and lincomycin (a lincosamide) both exhibit strong activity against a range of Gram-positive bacteria, including *S. pneumoniae* and S. aureus (38, 39). Their association with the shortest return time is highly consistent with the final diagnosis of *S. pneumoniae* bacterial conjunctivitis, providing supportive clinical outcome evidence for the etiological diagnosis. This suggests that agents with robust Gram-positive coverage may be more effective in this outbreak. Ofloxacin, a broad-spectrum fluoroquinolone, is typically highly active against *S. pneumoniae* (40). Its slightly longer associated return time, compared to tobramycin/lincomycin, may be attributable to local ocular pharmacokinetics, patient compliance, or chance variation within this study.

The epidemiological investigation pinpointed the field trip as the critical point of outbreak origin. While schools are well-established settings for respiratory and contact-transmitted disease outbreaks (20), this study broadens the risk context from static classrooms and cafeterias to dynamic, organized off-campus collective living environments. In contrast to routine school settings, field trips typically involve conditions that can significantly facilitate contact transmission: prolonged time in enclosed spaces (e.g., buses, dormitories); frequent physical collaboration and close contact during team-based activities; increased sharing of equipment, bedding, and washing facilities; and a potential relaxation of personal hygiene practices in a new environment. Collectively, these factors create an efficient environment for hand-eye and fomite-eye transmission. Although prior research has documented bacterial conjunctivitis outbreaks in collective living settings such as prisons (41), this study provides empirical support for the hypothesis that specific collective activity patterns can act as disease transmission accelerators by tracing a *S. pneumoniae* conjunctivitis outbreak to a primary school field trip and preliminarily outlining its transmission chain. The epidemic curve—a sharp rise in cases within days of the shared exposure, followed by a tail of secondary cases—is consistent with the pattern expected for a point-source exposure. Similar epidemic curves were observed in previous *S. pneumoniae* conjunctivitis outbreaks at a US college and an elementary school in Maine (42), where an initial common exposure was followed by sustained person-to-person spread.

This study has several limitations. First, as a retrospective investigation, it is possible that mild cases were missed, and recall bias regarding exposure details may have been introduced. Second, we acknowledge that we were unable to obtain cultured environmental isolates for genetic comparison with clinical isolates. Although environmental samples from the field trip base were PCR-positive for *S. pneumoniae*, all were culture negative, precluding molecular typing (e.g., whole-genome sequencing, PFGE, or MLST) to confirm strain identity between environmental and clinical sources. Consequently, the transmission pathway from the field trip environment to the students cannot be microbiologically confirmed at the strain level. This limitation reflects the inherent challenges of environmental sampling for fastidious organisms such as *S. pneumoniae*, which rapidly lose viability on desiccated surfaces, and the constraints of laboratory capacity during the emergency response. Nevertheless, the conclusion that the field trip was the outbreak source is strongly supported by a consistent body of epidemiological evidence: the case-control association (OR = 6.42, 95% CI: 3.53–11.68), the temporal sequence (index case onset immediately upon return), the spatial concentration of cases in the exposed grade (60.42% in fourth grade), environmental PCR positivity at the exposure site, and the successful culture of *S. pneumoniae* from clinical specimens. While a microbiological link would have provided definitive proof, the epidemiological chain of evidence is sufficiently robust to support the field trip as the outbreak source. Third, the observed association between combination antibiotic therapy and a shorter time to return to school was derived solely from observational analysis. Given the non-random allocation of treatment regimens, this finding carries a risk of confounding by indication. Additionally, individual-level demographic data for the control population were not accessible due to privacy restrictions imposed by the education authorities, which precluded multivariable logistic regression adjustment. However, given the strong effect size (OR = 6.42, 95% CI: 3.53–11.68), the consistency of findings across stratified analyses, and the robust temporal and spatial epidemiological evidence, we believe it is unlikely that unmeasured confounding by demographic factors could fully explain the observed association. Finally, as the conclusions are drawn from a single outbreak, their generalizability requires validation through the study of additional, similar events.

Despite its limitations, this outbreak offers vital public health warnings. Based on our findings, we recommend the following measures, which we have categorized according to the strength of evidence supporting them. Recommendations directly supported by this investigation: (1) Enhanced pre-activity health education specifically targeting hand hygiene, avoidance of eye-rubbing, and refraining from sharing personal items (e.g., towels, pillows) before and during off-campus group activities. This recommendation is directly supported by our case-control finding that field trip participation was the primary exposure. (2) On-site IPC support for collective activities, including ensuring adequate handwashing facilities and regular cleaning of high-touch surfaces (e.g., bus handrails, dormitory handles, shared equipment). This is supported by our environmental detection of *S. pneumoniae* DNA at the field trip dormitories, which identified the exposure site and implicated fomites in transmission. (3) CDC investigators should proactively inquire about recent off-campus group activities when investigating school-based conjunctivitis clusters. This recommendation is directly derived from our finding that the outbreak was traced to a specific field trip, a transmission setting not previously documented for *S. pneumoniae* conjunctivitis. In addition to the specific recommendations above, it is important to recognize the distinct roles that educators and school staff play in preventing and mitigating such outbreaks. First, the school principal or head serves as the first responsible person for infectious disease outbreak reporting and response, with responsibilities that include establishing reporting systems, coordinating with health authorities, and ensuring adequate resources for prevention measures. Second, homeroom teachers function as frontline sentinels; they are responsible for daily morning and noon health checks, monitoring student absenteeism, identifying early symptoms, and promptly reporting suspected cases to the school health officer or designated disease reporter. Third, school health personnel (school nurses or doctors) play a critical role in pre-trip health assessments for off-campus activities—including reviewing student health records, preparing emergency care plans, and training accompanying staff in medication administration and first aid—as well as providing infection prevention education. Fourth, accompanying teachers on field trips require specific training in infection prevention and control, including recognizing symptoms of infectious diseases, maintaining hygiene discipline among students (e.g., discouraging eye-rubbing and ensuring hand hygiene), managing minor incidents, and coordinating emergency response with the school nurse and principal. Finally, an integrated framework that clarifies these roles and provides regular IPC training is essential; scenario-based training programmes have been shown to significantly improve teachers' knowledge and practical competencies in infection prevention. By embedding these educational responsibilities into outbreak preparedness planning, schools can more effectively reduce transmission risks both on-campus and during off-campus group activities.

General infection prevention and control practices (applicable to all congregate settings): (1) Symptom surveillance and response mechanisms—training organizers and teachers to recognize early symptoms, establishing protocols for timely isolation of suspected cases, and ensuring medical referral. (2) Routine hand hygiene promotion and regular environmental disinfection in schools and group activity venues.

## 5 Conclusion

This study provides epidemiological evidence suggesting a link between an acute conjunctivitis outbreak caused by *S. pneumoniae* and a primary school educational field trip. Our observational data also suggest that combination antibiotic therapy was associated with shorter return-to-school time; however, this finding requires confirmation in controlled studies. Although the transmission pathway could not be microbiologically confirmed due to the inability to culture environmental isolates for genetic comparison, the consistent epidemiological, temporal, and environmental findings collectively support this conclusion. Given the relative rarity of this pathogen as an outbreak agent and the unique transmission context, these findings add to the existing literature on school-based conjunctivitis outbreaks. The results underscore the necessity of dynamically evaluating public health risks tied to increasingly varied educational activities and implementing proactive, targeted infection control measures to protect student health and ensure activity safety.

## Data Availability

The original contributions presented in the study are included in the article/Supplementary material, further inquiries can be directed to the corresponding authors.

## References

[1] Gui SY Qiao JC Wang XC Yang F Hu CY Tao FB . Long-term effects of meteorological factors and extreme weather on daily outpatient visits for conjunctivitis from 2013 to 2020: a time-series study in Urumqi, China. Environ Sci Pollut Res Int. (2023) 30:58041–57. doi: 10.1007/s11356-023-26335-4 36977878 PMC10047460

[2] Singh RB Liu L Anchouche S Yung A Mittal SK Blanco T . Ocular redness - I: etiology, pathogenesis, and assessment of conjunctival hyperemia. Ocul Surf. (2021) 21:134–44. doi: 10.1016/j.jtos.2021.05.003 34010701 PMC8328962

[3] Liu SH Hawkins BS Ng SM Ren M Leslie L Han G . Topical pharmacologic interventions versus placebo for epidemic keratoconjunctivitis. Cochrane Database Syst Rev. (2022) 3:CD013520. doi: 10.1002/14651858.CD013520.pub2 35238405 PMC8892837

[4] Kuo IC Gower EW. Cost savings from a policy to diagnose and prevent transmission of adenoviral conjunctivitis in employees of a large academic medical center. JAMA Ophthalmol. (2021) 139:518–24. doi: 10.1001/jamaophthalmol.2021.0150 33792644 PMC8017479

[5] Yan D Prajna NV Lalitha P Sansanayudh W Satitpitakul V Laovirojjanakul W . Association of weather variables with pathogens contributing to conjunctivitis worldwide. Clin Infect Dis. (2025) 80:551–61. doi: 10.1093/cid/ciae417 39158989 PMC11912971

[6] Johnson D Liu D Simel D. Does this patient with acute infectious conjunctivitis have a bacterial infection? The Rational Clinical Examination systematic review. JAMA. (2022) 327:2231–7. doi: 10.1001/jama.2022.7687 35699701

[7] Ching SJ Jung GO Osuna A Casey T Xia H Bostwick K . Outbreak of Neisseria meningitidis conjunctivitis in military trainees - Texas, February-May 2025. MMWR Morb Mortal Wkly Rep. (2025) 74:516–21. doi: 10.15585/mmwr.mm7433a1 40906586 PMC12410586

[8] Azari AA Barney NP. Conjunctivitis: a systematic review of diagnosis and treatment. JAMA. (2013) 310:1721–9. doi: 10.1001/jama.2013.280318 24150468 PMC4049531

[9] Muto T Imaizumi S Kamoi K. Viral conjunctivitis. Viruses. (2023) 15:676. doi: 10.3390/v15030676 36992385 PMC10057170

[10] Seo JW Lee SK Hong IH Choi SH Lee JY Kim HS . Molecular epidemiology of adenoviral keratoconjunctivitis in Korea. Ann Lab Med. (2022) 42:683–7. doi: 10.3343/alm.2022.42.6.683 35765877 PMC9277046

[11] Philomenadin FS Singh MP Shastri J Phukan AC Nagarajan M Kaliaperumal S . Whole genome analysis of Human Mastadenovirus D causing keratoconjunctivitis in India - a multicentre study. J Infect Dev Ctries. (2024) 18:450–7. doi: 10.3855/jidc.18905 38635622

[12] Chavan NA Rani VS Shinde P Shinde M Pavani S Srinath M . Identification of coxsackievirus A-24 GIV C5 strain as the cause of acute hemorrhagic conjunctivitis outbreak in Hyderabad, India in 2022. Heliyon. (2024) 10:e32254. doi: 10.1016/j.heliyon.2024.e32254 38947457 PMC11214445

[13] Johansson E Caraballo R Hurdiss DL Mistry N Andersson CD Thompson RF . Exploring the effect of structure-based scaffold hopping on the inhibition of coxsackievirus A24v transduction by pentavalent N-acetylneuraminic acid conjugates. Int J Mol Sci. (2021) 22:8418. doi: 10.3390/ijms22168418 34445134 PMC8395083

[14] Hu YL Lee PI Hsueh PR Lu CY Chang LY Huang LM . Predominant role of Haemophilus influenzae in the association of conjunctivitis, acute otitis media and acute bacterial paranasal sinusitis in children. Sci Rep. (2021) 11:11. doi: 10.1038/s41598-020-79680-6 33420151 PMC7794412

[15] Afzal M Vijay AK Stapleton F Willcox M. Virulence genes of Staphylococcus aureus associated with keratitis, conjunctivitis, and contact lens-associated inflammation. Transl Vis Sci Technol. (2022) 11:5. doi: 10.1167/tvst.11.7.5 35802366 PMC9279920

[16] Saberinia A Ozmaei S Anoushepour A. Tear film osmolarity in horses with bacterial conjunctivitis. Vet Med Sci. (2025) 11:e70677. doi: 10.1002/vms3.70677 41243880 PMC12620991

[17] Martin M Turco JH Zegans ME Facklam RR Sodha S Elliott JA . An outbreak of conjunctivitis due to atypical Streptococcus pneumoniae. N Engl J Med. (2003) 348:1112–21. doi: 10.1056/NEJMoa022521 12646668

[18] Buck JM Lexau C Shapiro M Glennen A Boxrud DJ Koziol B . A community outbreak of conjunctivitis caused by nontypeable Streptococcus pneumoniae in Minnesota. Pediatr Infect Dis J. (2006) 25:906–11. doi: 10.1097/01.inf.0000238143.96607.ec 17006286

[19] Leung AKC Hon KL Wong AHC Wong AS. Bacterial conjunctivitis in childhood: etiology, clinical manifestations, diagnosis, and management. Recent Pat Inflamm Allergy Drug Discov. (2018) 12:120–7. doi: 10.2174/1872213X12666180129165718 29380707

[20] Endo A Uchida M Hayashi N Liu Y Atkins KE Kucharski AJ . Within and between classroom transmission patterns of seasonal influenza among primary school students in Matsumoto city, Japan. Proc Natl Acad Sci U S A. (2021) 118:e2112605118. doi: 10.1073/pnas.2112605118 34753823 PMC8609560

[21] Zivich PN Eisenberg MC Monto AS Uzicanin A Baric RS Sheahan TP . Transmission of viral pathogens in a social network of university students: the eX-FLU study. Epidemiol Infect. (2020) 148:e267. doi: 10.1017/S0950268820001806 32792023 PMC7689784

[22] Banholzer N Munday JD Jent P Bittel P. Dall'Amico L, Furrer L,et al. The relative contribution of close-proximity contacts, shared classroom exposure and indoor air quality to respiratory virus transmission in schools. Nat Commun. (2025) 16:11678. doi: 10.1038/s41467-025-66719-3 41309642 PMC12749238

[23] Kratzer S Pfadenhauer LM Biallas RL Featherstone R Klinger C Movsisyan A . Unintended consequences of measures implemented in the school setting to contain the COVID-19 pandemic: a scoping review. Cochrane Database Syst Rev. (2022) 6:CD015397. doi: 10.1002/14651858.CD015397 35661990 PMC9169532

[24] Kim SJ Kim EY Yu J. Characteristics of a large outbreak arising from a school field trip after COVID-19 restrictions were eased in 2022. Osong Public Health Res Perspect. (2024) 15:83–9. doi: 10.24171/j.phrp.2023.0264 38481052 PMC10982658

[25] Thomas CM Foster A Boop S Kirschke D Mooney H Reid I . Shiga toxin-producing Escherichia coli O157:H7 outbreak associated with school field trips at a farm animal exhibit-Tennessee, September-October 2023. Zoonoses Public Health. (2024) 71:829–35. doi: 10.1111/zph.13161 38858856 PMC11951936

[26] Zivich PN Grabenstein JD Becker-Dreps SI Weber DJ. Streptococcus pneumoniae outbreaks and implications for transmission and control: a systematic review. Pneumonia. (2018) 10:11. doi: 10.1186/s41479-018-0055-4 30410854 PMC6217781

[27] Matthews AJ Rowe HM Rosch JW Camilli A A. Tn-seq screen of *streptococcus pneumoniae* uncovers DNA repair as the major pathway for desiccation tolerance and transmission. Infect Immun. (2021) 89:e0071320. doi: 10.1128/IAI.00713-20 34031124 PMC8281258

[28] Lane JR Tata M Yasmin R Im H Briles DE Orihuela CJ. PspA-mediated aggregation protects Streptococcus pneumoniae against desiccation on fomites. bioRxiv. (2023) 30:2023.09.27.559802. doi: 10.1128/mbio.02634-23 37982608 PMC10746202

[29] Rousseau A Resnikoff S Vauloup-Fellous C Loukil M Barreau E Zina S . Conjonctivites virales et chlamydiennes [Viral and chlamydial conjunctivitis]. J Fr Ophtalmol. (2024) 47:104337. doi: 10.1016/j.jfo.2024.104337 39454485

[30] Dagan R Ben-Shimol S Greenberg D Givon-Lavi N A. prospective, population-based study to determine the incidence and bacteriology of bacterial conjunctivitis in children < 2 years of age following 7-valent and 13-valent pneumococcal conjugate vaccine sequential implementation. Clin Infect Dis. (2021) 72:1200–7. doi: 10.1093/cid/ciaa197 32140705

[31] Siegel H Lang S Maier P Reinhard T. Bakterielle Konjunktivitis: Aktuelle Aspekte der Diagnostik und Therapie [Bacterial conjunctivitis: current aspects of diagnosis and therapy]. Klin Monbl Augenheilkd. (2024) 241:231–46. doi: 10.1055/a-2193-2658 37977204

[32] Lv G Shi L Liu Y Sun X Mu K. Epidemiological characteristics of common respiratory pathogens in children. Sci Rep. (2024) 14:16299. doi: 10.1038/s41598-024-65006-3 39009660 PMC11251276

[33] Mohamed YH Toizumi M Uematsu M Nguyen HT Le LT Takegata M . Prevalence of Streptococcus pneumoniae in conjunctival flora and association with nasopharyngeal carriage among children in a Vietnamese community. Sci Rep. (2021) 11:337. doi: 10.1038/s41598-020-79175-4 33431887 PMC7801475

[34] Parker AM Jackson N Awasthi S Kim H Alwan T Wyllie AL . Upper respiratory Streptococcus pneumoniae colonization among working-age adults with prevalent exposure to overcrowding. Microbiol Spectr. (2024) 12:e0087924. doi: 10.1128/spectrum.00879-24 39012111 PMC11302326

[35] Hu T Chu D Cui H Wang S Wang M Liang R . Zwitterionic chitosan-modified amorphous ZnCuAl-LDH eye drops for efficient treatment of bacterial keratitis. Mater Today Bio. (2025) 33:101998. doi: 10.1016/j.mtbio.2025.101998 40600178 PMC12212269

[36] Shekhawat NS Shtein RM Blachley TS Stein JD. Antibiotic prescription fills for acute conjunctivitis among enrollees in a large United States managed care network. Ophthalmology. (2017) 124:1099–107. doi: 10.1016/j.ophtha.2017.04.034 28624168 PMC9482449

[37] Kowalski RP Yates KA Romanowski EG Karenchak LM Mah FS Gordon YJ. An ophthalmologist's guide to understanding antibiotic susceptibility and minimum inhibitory concentration data. Ophthalmology. (2005) 112:1987. doi: 10.1016/j.ophtha.2005.06.025 16183128

[38] Austin MS. In vivo intra-articular antibiotic concentrations at 24 hours after TKA fall below the minimum inhibitory concentration for most bacteria: a randomized study of commercially available bone cement. J Bone Joint Surg Am. (2024) 106:1664–72. doi: 10.2106/JBJS.23.01412 39052763

[39] Mukai K Shibayama T Imai Y Hosaka T. Phenomenological interpretations of the mechanism for the concentration-dependent positive effect of antibiotic lincomycin on Streptomyces coelicolor A3. Appl Environ Microbiol. (2023) 89:e0113323. doi: 10.1128/aem.01133-23 37732750 PMC10617593

[40] Najm MAA Shakir HA Hasen ST Jawad KH Hasoon BA Jabir MS . Titanium dioxide nanoparticles augment ciprofloxacin activity via inhibition of biofilm formation for multidrug resistance bacteria in-vitro and insilco prediction study. Sci Rep. (2025) 15:18014. doi: 10.1038/s41598-025-93569-2 40410449 PMC12102197

[41] Sanchez GV Bourne CL Davidson SL Ellis M Feldstein LR Fay K . Pneumococcal disease outbreak at a state prison, Alabama, USA, September 1–October 10, 2018. Emerg Infect Dis. (2021) 27:1949–52. doi: 10.3201/eid2707.203678 34152958 PMC8237874

[42] Centers for Disease Control and Prevention (CDC). Pneumococcal conjunctivitis at an elementary school–Maine, September 20-December 6, 2002. MMWR Morb Mortal Wkly Rep. (2003) 52:64-6. 12578323

